# Genistein induces macrophage polarization and systemic cytokine to ameliorate experimental colitis

**DOI:** 10.1371/journal.pone.0199631

**Published:** 2018-07-19

**Authors:** Jessicca D. Abron, Narendra P. Singh, Robert L. Price, Mitzi Nagarkatti, Prakash S. Nagarkatti, Udai P. Singh

**Affiliations:** 1 Pathology, Microbiology and Immunology, School of Medicine, University of South Carolina, Columbia, SC; 2 Cell Biology and Anatomy, School of Medicine, University of South Carolina, Columbia, SC, United States of America; Toho Daigaku, JAPAN

## Abstract

Mucosal changes in Crohn’s disease (CD) and ulcerative colitis (UC), two major forms of inflammatory bowel disease (IBD), are characterized by a prominent infiltration of inflammatory cells including lymphocytes, macrophages, T cells and neutrophils. The precise etiology of IBD is unknown but it involves a complex interplay of factors associated with the immune system, environment, host genotype and enteric commensal bacteria. As there is no known safe cure for IBD, natural alternative therapeutic options without side effects are urgently needed. To this end, Soy-based foods, which have been eaten for centuries in Asian countries, have potential benefits, including lowering the incidence of coronary heart disease, atherosclerosis, type-2 diabetes, allergic response, and autoimmune diseases. This study describes the effect of Soy isoflavons 4', 5, 7 Trihydroxyisoflavone (genistein) on dextran sodium sulphate (DSS) induced experimental colitis. The extent and severity of disease was analyzed through body weight, histopathological analysis, cellular immune response, systemic cytokine levels, and inflammation score using a disease activity index. Genistein treatment significantly attenuated DSS-induced colitis severity and resulted in increase in body weight, colon length and reduction in inflammation score. Genistein also skews M1 macrophages towards the M2 phenotype. Further, gen also reduced the systemic cytokine levels as compared to vehicle control. This serves as the first detailed study towards natural soya based product that shows the polarization of M1 towards M2 macrophages, and reduction of systemic cytokine in part to attenuate the colitis symptoms. Thus, our work demonstrates that genistein, a soya compound, may be useful for the treatment of IBD.

## Introduction

Chronic inflammatory bowel diseases (IBD) such as Crohn’s disease (CD) and ulcerative colitis (UC) are a serious threat to all corners of world. Approximately 2.3 million European and about 1.4 million peoples suffer from IBD in USA [1]. Although the mechanism of IBD has been under investigation for more than half a century, the etiology is unknown and more efficient therapies are still needed [2]. Mucosal changes in IBD are characterized by prominent infiltration of various cells including T lymphocytes, neutrophils and macrophages. Although IBD is maintained by T helper cells/macrophage-driven immune responses, the overall mechanism responsible for IBD is believed to involve a complex interplay between a plethora of inflammatory mediators. To this end, macrophages, which are an essential part of the innate immune system, neutrophils and T cells, have received significant attention in the development of experimental colitis and IBD.

Macrophages have important functions in mediating host defense mechanisms against infection and inflammation [3]. As prominent effector cells of both innate and adaptive immune responses, macrophages undoubtedly provide important protection against harmful local antigens such as those that cause intestinal inflammation. In tissues, macrophages are activated and produce multiple cytokines in response to various signals and change to classical M1 (pro-inflammatory) or M2 (anti-inflammatory) phenotypes. M1 macrophages produce high levels of IL-12 and IL-23 and M2 macrophages are marked by the expression of arginase-1 (ARG-1), IL-10 and IL-13 that has an immunoregulatory function [4, 5]. Macrophages are present in distinctly elevated numbers in the mucosa of both CD and UC patients [6]. Thus, understanding the underlying mechanism of activation and different phenotypes of macrophages during intestinal inflammation is important for the design of therapeutic approaches.

Clinical studies have shown that soy-based foods, which have been eaten for centuries in Asian countries, have potential benefits, including lowering the incidence of coronary heart disease, atherosclerosis, and type-2 diabetes [7–9]. Soy isoflavons 4', 5, 7 Trihydroxyisoflavone (Genistein) have been shown to be beneficial in antigen-immunized, allergic, and autoimmune models [10–12]. Soy is also an important mediator of gastrointestinal inflammation [13] and acute colitis. Genistein can bind estrogen receptors (ERs) predominantly expressed in the gastrointestinal tract and it is possible that macrophages that express ERs can mediate inflammatory modulation and reduce intestinal inflammation.

In the present study, we investigated the effect of genistein, commonly known as soya, in ameliorating colon inflammation in DSS induced experimental colitis. We examined the implications of genistein on macrophage frequency, phenotype (M1 and M2), cytokines and Th17 cells known to play prominent roles in colitis development. The results from this study clearly suggest that genistein protects mice from DSS induced colitis, which may be due to polarization of M1 macrophages towards M2 phenotype and at least in part by reduction in the systemic proinflammatory cytokines.

## Materials and methods

### Animals

Female wild-type C57BL/6 mice, aged 8 to 12 weeks, were purchased from Jackson Laboratories (Bar Harbor, ME). The animals were housed in the University of South Carolina’s School of Medicine Animal Facility. All mice were maintained in an isolator cages under normal light and dark cycles in conventional housing conditions in an attempt to minimize animal pain and distress. We observed mice twice daily (morning and evening) for pain and distress. Towards this, we monitored the mice mobility, licking, biting, scratching or shaking a particular area, abnormal resting postures, diarrhea and blood in fecal matter. If we noticed any of these symptoms and loss of body weight more than 18%, mice was received Ibuprofen (40 mg/kg) in water bottle. If mice lost more than 22% body weight and slow in movement, was euthanized and removed from the experiment. At the end of experiment, mice was scarified by Isoflurane gas anesthesia using a two compartment glass jar, putting mice on upper chamber to avoid direct contact with anesthesia in lower chamber. Experimental groups consisted of 6 female mice and studies were repeated 3 times (total N = 18). The University of South Carolina's Institutional Animal Care and Use Committee approved all animal experimentation used in this study.

### Experimental colitis induced by dextran sodium sulphate (DSS)

Experimental colitis was induced using DSS as described in our previously published work [14]. Briefly, eight week-old C57BL/6 mice received either normal water or water containing 3% DSS (MP Biomedical, LLC, Ohio Molecular weight 36, 000–50,000) (*ad libitum)* for a cycle of seven day followed by seven days cycle of normal water totaling of 14 days. Body weight of mice was monitored every day following the induction of DSS. In addition, other sickness symptoms including diarrhea and blood in fecal matter were monitored during this time. At the end of the experimental period blood was collected and colons were washed with phosphate-buffered saline (PBS), cut longitudinally, formalin fixed, and embedded in paraffin.

### Genistein treatment

Genistein was purchased from Tocris Bioscience, Minneapolis, MN (more than 98% pure by HPLC). In a pilot study, we found that the 10mg/kg body weight dose of genistein used in this study was more efficacious in reducing colitis progression than were other tested doses (5, 20, 40, 100 mg/kg). Therefore, we used this dose in present study. Mice were then given either 100 μl (10 mg/kg body weight) of genistein solution or vehicle (PBS) by oral gavage every day till days 14. Since, we did not notice any major changes in body weight of naïve and genistein alone treated mice, thus data from these groups are not included in this study. Therefore, we compare the changes between vehicle and genistein treated mice after DSS induction.

### Cell isolation from systemic and mucosal organs

At the experimental end point on day 14, spleens and mesenteric lymph nodes (MLNs) from individual mice of all groups were dissociated and red blood cells (RBCs) lysed using lysis buffer (Sigma, St. Louis, MO). Single-cell suspensions of spleen and MLNs were passed through a sterile filter (Sigma, St. Louis, MO) to remove any debris. Subsequently, cell suspensions were washed twice in RPMI 1640 (Sigma, St. Louis, MO) and stored in media containing 3% fetal bovine serum (FBS) on ice or at 4°C until used on the same day. Cells from the colon lamina propria (cLP) were isolated as described previously [15]. In brief, colon was cut into small strips and stirred in PBS containing 1mM EDTA at 37°C for 30 min. The colon tissue was digested with collagenase type IV (Sigma St. Louis, MO) in RPMI 1640 (collagenase solution) for 45 min at 37°C with moderate stirring. After each 30 min interval, the released cells were centrifuged and stored in complete media containing 3% FBS. Colon pieces were again treated with fresh collagenase solution, at least two times, and cells were then pooled. The cells from cLP were further purified using a discontinuous Percoll (Pharmacia, Uppsala, Sweden) gradient collecting at the 40–75% interface. Lymphocytes were maintained in complete medium as described in detail in our earlier publications [14, 16].

### Flow cytometry staining and analysis

Cells from the spleen, MLNs, and cLP were isolated as described above for four experimental groups. Since, we did not notice any major changes in Naïve as well as genistein treatment alone, flow data is not included for these groups in this manuscript. The cells were washed with FACS staining buffer (PBS with 1% FBS), and then stained with the manufacturer’s suggested concentration of FITC-or APC-conjugated anti-CD4 (GK1.5) (Biolegend, San Diego, USA), FITC conjugated CD11b (M1/70) (BD-PharMingen, San Diego, CA), APC conjugated APC rat anti-mouse IL-10 (JES5-16E3) (BD-PharMingen, San Diego, CA), FITC conjugated mouse anti- ARG-1 (BD Transduction Laboratories), PE conjugated anti-mouse CD11c (HL3) (BD-PharMingen, San Diego, CA), FITC-conjugated F4/80 (BM8) and PE-conjugated CD206 (C068C2) Biolegend (San Diego, CA), for 30 minutes with occasional shaking at 4°C. The cells were washed two times with FACS staining buffer and thoroughly re-suspended in BD Cytofix/Cytoperm (BD-PharMingen, San Diego CA) solution for 20 min. The cells were again washed two times with BD perm/wash solution after storage for 10 min at 4°C. Cells were then washed thoroughly with FACS staining buffer and analyzed by flow cytometry (FC 500/BD FACS Aria II by Beckman Coulter/ BD Fort Collins CO/San Diego CA).

### Dendritic (DCs), CD4 T+IL-10^+^ cells frequency and M2 phenotypes characterization

M2 cells based on F4/80^+^ CD206^+^ staining obtained from vehicle and genistein treated DSS induced mice were sort-purified using a FACS-Aria (Becton Dickinson) (purity >94%). These cells were stained for the expression ARG-1 and analyzed by flow cytometry. Further, these purified M2 cells were cultured alone at 37°C in 5% CO_2_ for 24 hours to determine the IL-10 level in culture supernatant. The flow cytometry percentage data of both DCs and IL-10 producing CD4^+^ T cells were enumerated by calculating total number of cells obtained from experiment from each mucosal organ (N = 18 for spleen, MLN and cLP) after vehicle and genistein treatment.

### Systemic cytokine measurement by luminex analysis

Levels of T helper cell-derived cytokines IL-6, TNF-α, IL-10, IL-1β and MCP-1were determined in the serum using a luminex Elisa assay kit (Bio Rad, Hercules, USA). In brief, IL-6, IL-10, TNF-α, IL-1β and MCP-1 analyte beads contained in assay buffer were added to pre-wet vacuum wells followed by 50 μl of assay beads. The buffer was then removed and the wells underwent a wash cycle. Next, 50 μl of standard or serum was added to each well and the plate was incubated for 1 hour and subjected to continuous shaking (at setting #3) using a Lab-Line™ Instrument Titer Plate Shaker (Melrose, IL). The filter bottom plates were then washed and vortexed at 300x g for 30 seconds. Subsequently, 25 μl of anti-mouse detection Ab was added to each well and incubated for 30 minute at room temperature (RT). Next, 50 μl of streptavidin-phycoerythrin solution was added and the plate was again incubated with continuous shaking for 10 minutes at RT. Finally, 125μl of assay buffer was added and BioRad readings were measured using a Luminex™ System (Austin, TX) and calculated using BioRad software. The Ab BioRad MAP assays are capable of detecting >10 pg/ml for each analytes.

### Histology

The colon was preserved using 4% paraformaldehyde prior to being embedded in paraffin. Fixed tissues were sectioned at 6 μm, and stained with hematoxylin and eosin for microscopy examination. Colon sections were graded according to the number of lesions as well as their severity of disease. A score (0 to 12) was given based on previously established criteria from our laboratory [15]. The summation of scores provided a total colonic disease score per mouse.

### Statistics

Statistical analysis was performed using one-way ANOVA with a two-tailed student's *t*-test using XLStat software (Addinosoft, Brooklyn, NY, USA). The results were further analyzed using the Statview II statistical program (Abacus Concepts, Inc., Berkeley, CA) for Macintosh computers. The change in body weight, colon length and inflammation score was measured longitudinally for the two groups, which depicts the mean ± the standard error of the sample mean (SEM). The infiltration of immune cell comparisons in the two groups was analyzed using ANOVA. Comparisons using ANOVA were also performed with respect to all flow cytometry studies. Statistical significance was assessed at the 5% level of significance, when the p-value was < 0.05 and indicated by the symbol.

## Results

### Genistein diminishes the severity of intestinal inflammation

**Fig 1:**
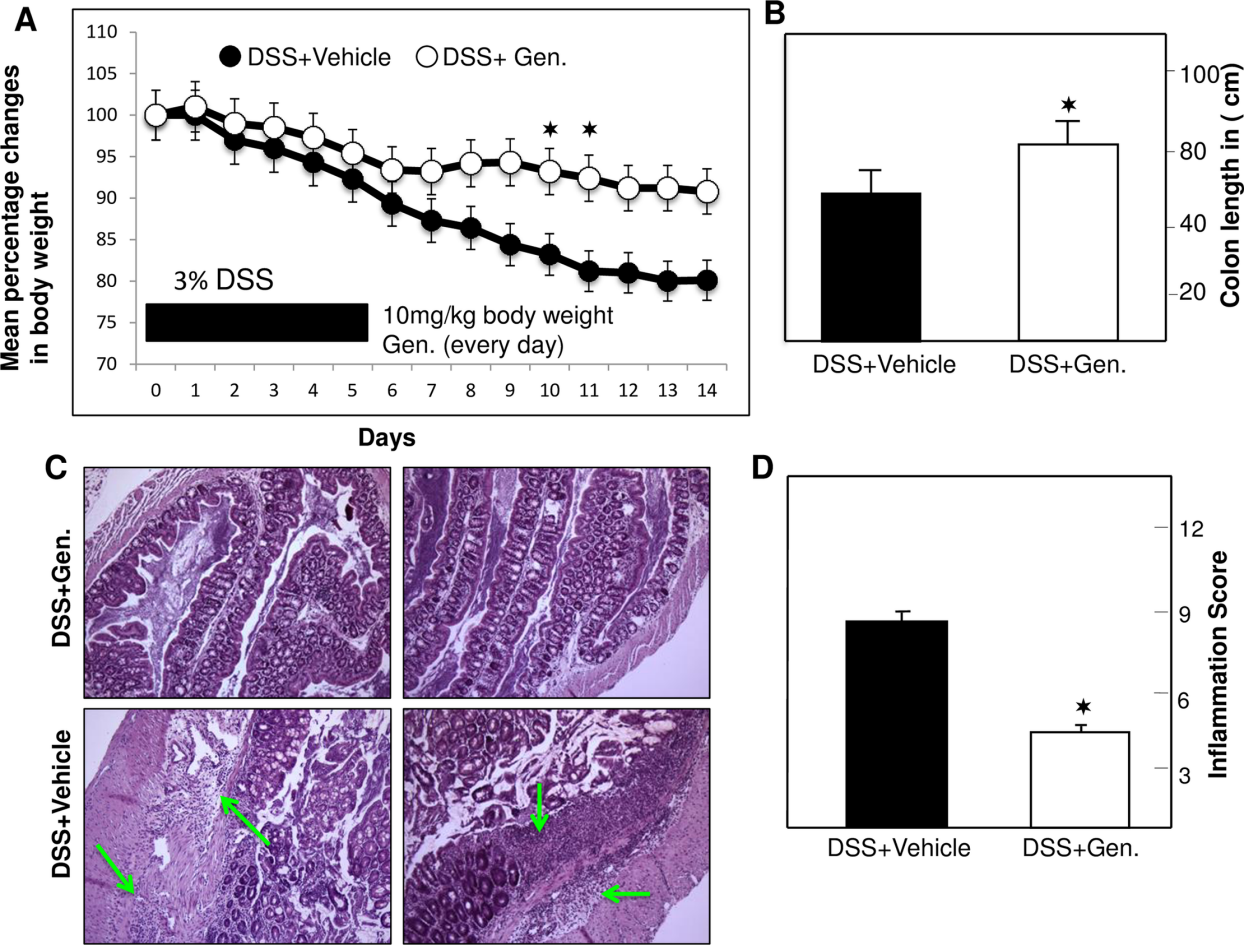
Genistein treatment mediates body weight, severity of inflammation, colon length and inflammation score in experimental colitis. Panel (A) BL/6 mice, received 100 μl of Vehicle+ DSS (●) and Genistein+ DSS (○) until day 14 after 3% DSS induction in water. Body weight was recorded every day in the morning hours. The change from initial body weight was expressed as a percentage change in body weight. Panels B and D: change in colon length and inflammation score in vehicle and genistein treated groups. Panel C: DSS induced vehicle treated mice show significant cellular infiltration and distortion of glands, while genistein treated mice (lower panel) showed markedly reduced cellular infiltration. The pathologic changes included diffuse cellular infiltrates, and thickening of the LP in the area of distorted crypts. Representative sections are shown from three separate experiments (20X magnification) containing six mice in each group. The statistical significance (✶) P<0.05 between vehicle controls values from genistein versus vehicle treated mice were assessed using Student’s t-test. Data represent the mean of three independent experiments involving six mice per group ± SEM.

Studies from the past suggest that a reduction in body weight is the hallmark of disease progression in colitis. Therefore, we recorded the body weight of mice every day in the morning. DSS induced mice given PBS as vehicle showed signs of inflammation, including continuous weight loss up to ~15% of their initial body weight (Fig 1A). In contrast, the body weight of genistein-treated mice increased following initial decline as compared to that of vehicle-treated mice (Fig 1A). Colon length also increased in mice that received genistein treatment as compared to vehicle treatment (Fig 1B). Increases in cellular infiltration (Fig 1C lower panel) and distortion of crypts were observed in colons after DSS induction. As compared with mice given vehicle, genistein treated mice showed reductions in inflammation and cellular infiltration (arrow), as well as partially restored glandular cell architecture (Fig 1C upper panel). Further, we assessed whether these effects can reverse inflammation scores in the colon, and found that the mean histological scores of mice with colitis treated with genistein were significantly lower than those of mice given vehicle (Fig 1D). Together, these results suggest that genistein attenuates colon inflammation associated with colitis.

### T cell response after DSS induction

**Fig 2:**
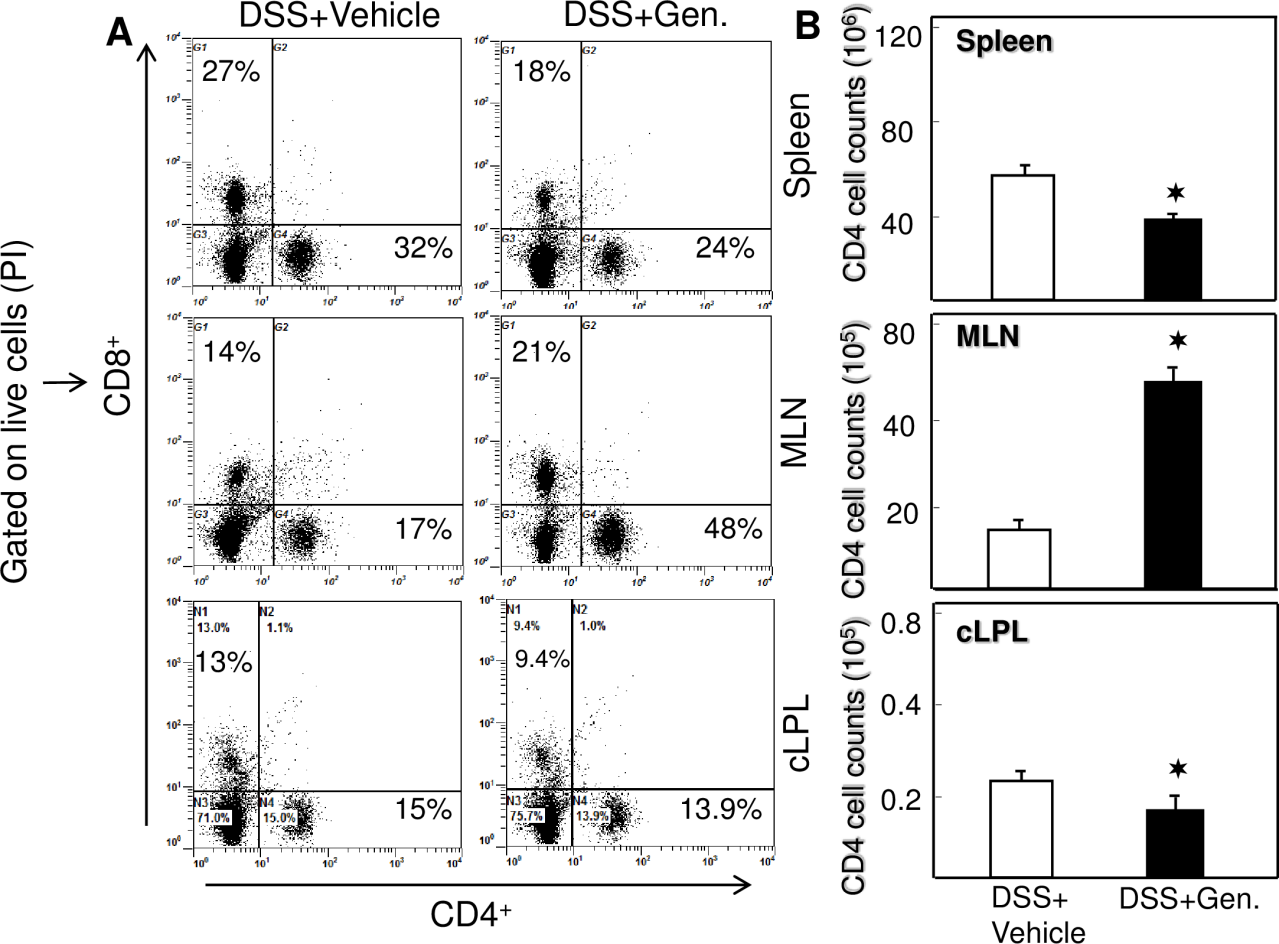
Genistein treatment facilitates the percentage and number of T helper cells. Splenic, MLN and LP lymphocytes were isolated from the two groups of mice as described in Fig 1 stained for CD4 and CD8 cell markers, and analyzed by using flow cytometry. The numbers in the upper left quadrant of each FACS chart indicate the total percentage of CD8 and those in the lower right quadrant of CD4 T cells. Data from a representative of three independent experiments are shown.

DSS can influence epithelial cell damage and induces proinflammatory cytokines to induce T- helper cell responses is believed to be a hallmark for colitis progression. Therefore, we examined T cell populations in the spleen, MLNs and cLP using flow cytometry analysis. The percentage of both T cells (CD4 and CD8) increased in the spleen and cLP of wild-type mice that received DSS induction with vehicle (PBS) as compared to mice that received DSS with genistein (Fig 2). In contrast, after DSS exposure, the percentage and number of T cells in MLNs was reduced significantly, but the percentage and number increased after genistein treatment. Thus, these results clearly suggest that genistein mediates T cells from systemic site and colon to reduces inflammation, while inducing more T cells at inductive site (MLNs) for expansion due to DSS exposure.

### Genistein impairs M1 macrophages associated with colitis

**Fig 3:**
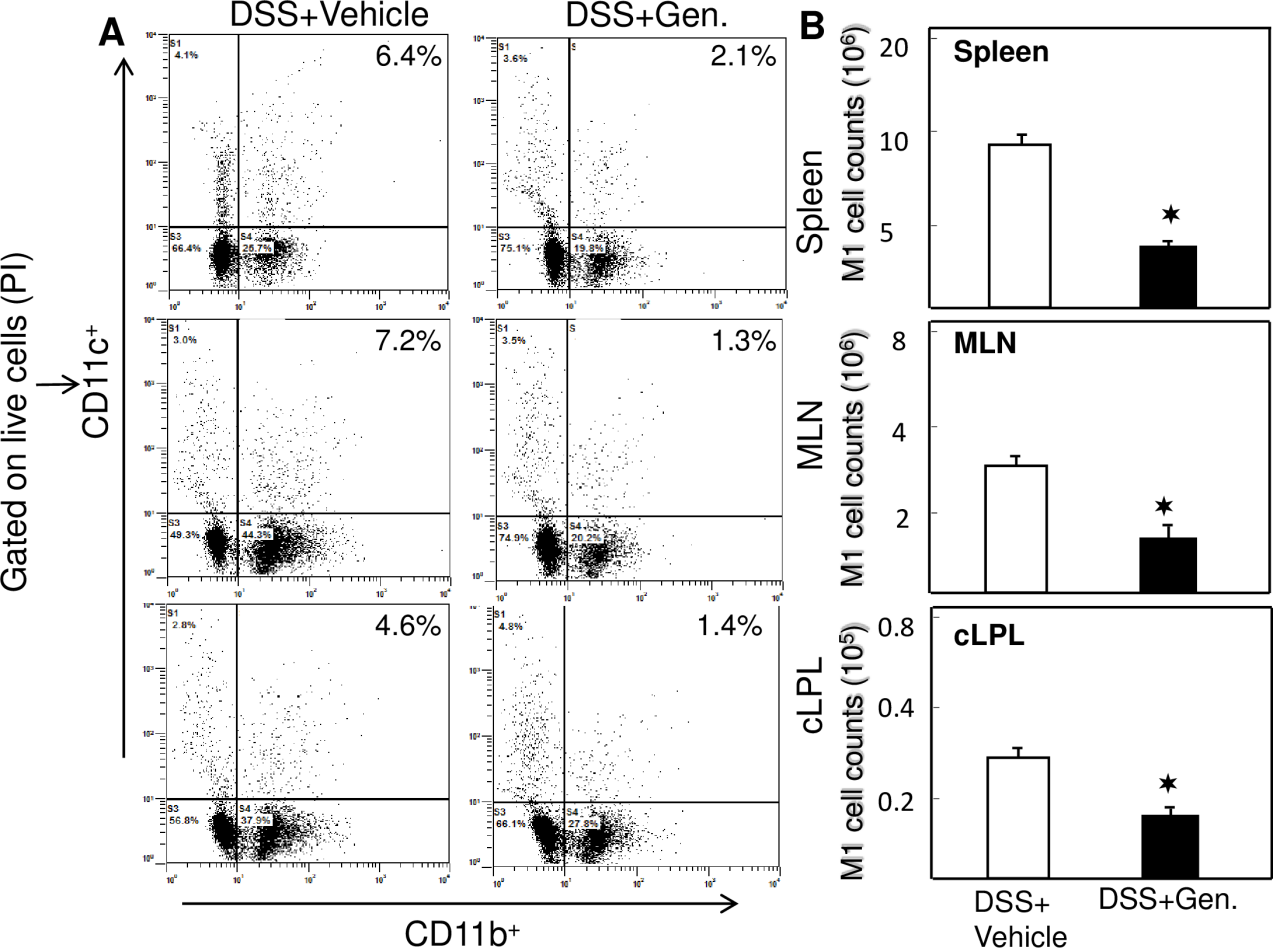
Genistein decreases the percentage and number of M1 macrophages. Genistein reduces the number and percentage of M1 macrophages in mice with acute colitis. Splenic, MLNs, and LP lymphocytes were isolated from the two groups of mice. Changes in the mean percentage of CD11b + CD11c (M1 phenotype) expression were compared between groups. Numbers in the upper right quadrant of the charts indicate the total percentage of M1 macrophages phenotypes. Data represent the total frequency of cells ± SEM from three independent experiments. Asterisks (✶) indicate statistically significant differences (p < 0.01) between vehicle-and genistein-treated groups.

It has been well established that M1 macrophages contribute critically to DSS induced colitis [17] through induction of multiple cytokines. Compared to DSS induced mice, the percentage of both mucosal and systemic M1 macrophages (CD11b+CD11C) decreased in the spleen, MLNs and cLP of genistein treated mice (Fig 3A). We also enumerated the numbers of M1 expressing macrophages from spleen, MLNs and cLP cells. To this end, we observed a significant decline in the number of M1 macrophages in the spleen, MLNs, and cLP of genistein treated as compared to vehicle treated mice (Fig 3B). These data indicate that impaired mucosal and systemic M1 responses resulting from DSS induction are associated with the impediment of colitis development. The percentage and number of M1 macrophages increased at mucosal sites to induce colitis, while genistein reduced both number and percentage of M1 macrophages to lessen the colitis symptoms.

### Genistein induced M2 macrophages phenotypes

**Fig 4:**
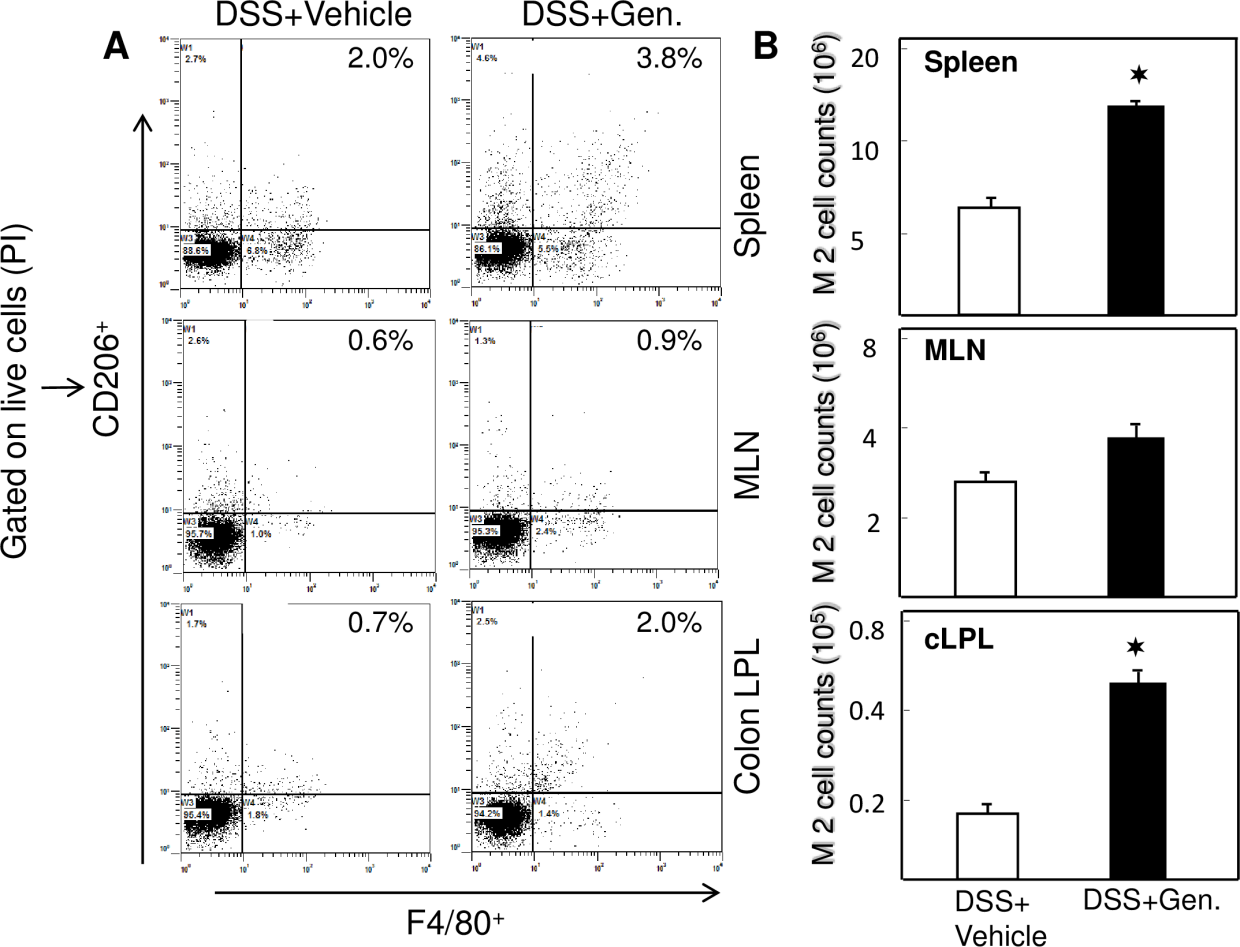
Genistein increases the percentage and number of M2 macrophage phenotypes. Genistein induces increased M2 macrophage percentage in mice with colitis. Splenic, MLN, and LP lymphocytes were isolated from the two groups of mice. Changes in the mean percentage of F4/80+CD206 (M2 phenotypes) expression were compared between two groups. Numbers in the upper right quadrant indicate the total percentage of M2 phenotypes. Data represent the total percentage of cells ± SEM from three independent experiments. Asterisks (✶) indicate statistically significant differences (p < 0.01) between vehicle- and genistein-treated groups.

Inflammatory macrophages massively infiltrate in the mucosa, clear wounds from bacteria and cellular debris, and wound-healing M2 macrophages promote tissue remodeling and maintain gut homeostasis. Macrophages carrying the mannose receptor CD206 are considered wound-healing macrophages and increased numbers found in the injured mucosa of UC patients [18]. Based on this, we next determined the changes in M2 macrophage (F4/80+CD206) percentage and number in response to genistein treatment after DSS induction. As hypothesized, DSS induced mice showed decreased percentage of M2 macrophages in the spleen, MLNs and cLP (Fig 4). However, we did notice a significant increase (P < 0.05) in M2 population in genistein treated mice as compared to vehicle treated DSS induced mice. We also noticed similar changes in the number of M2 macrophages in spleen and cLP in the genistein treated group as compared to vehicle group. These data imply that genistein treated mice show an increase in M2 macrophages after DSS induction, which suggests a possible beneficial mechanism for the observed decrease in severity of colitis in these mice.

### Systemic cytokine level changes after genistein treatment

**Fig 5:**
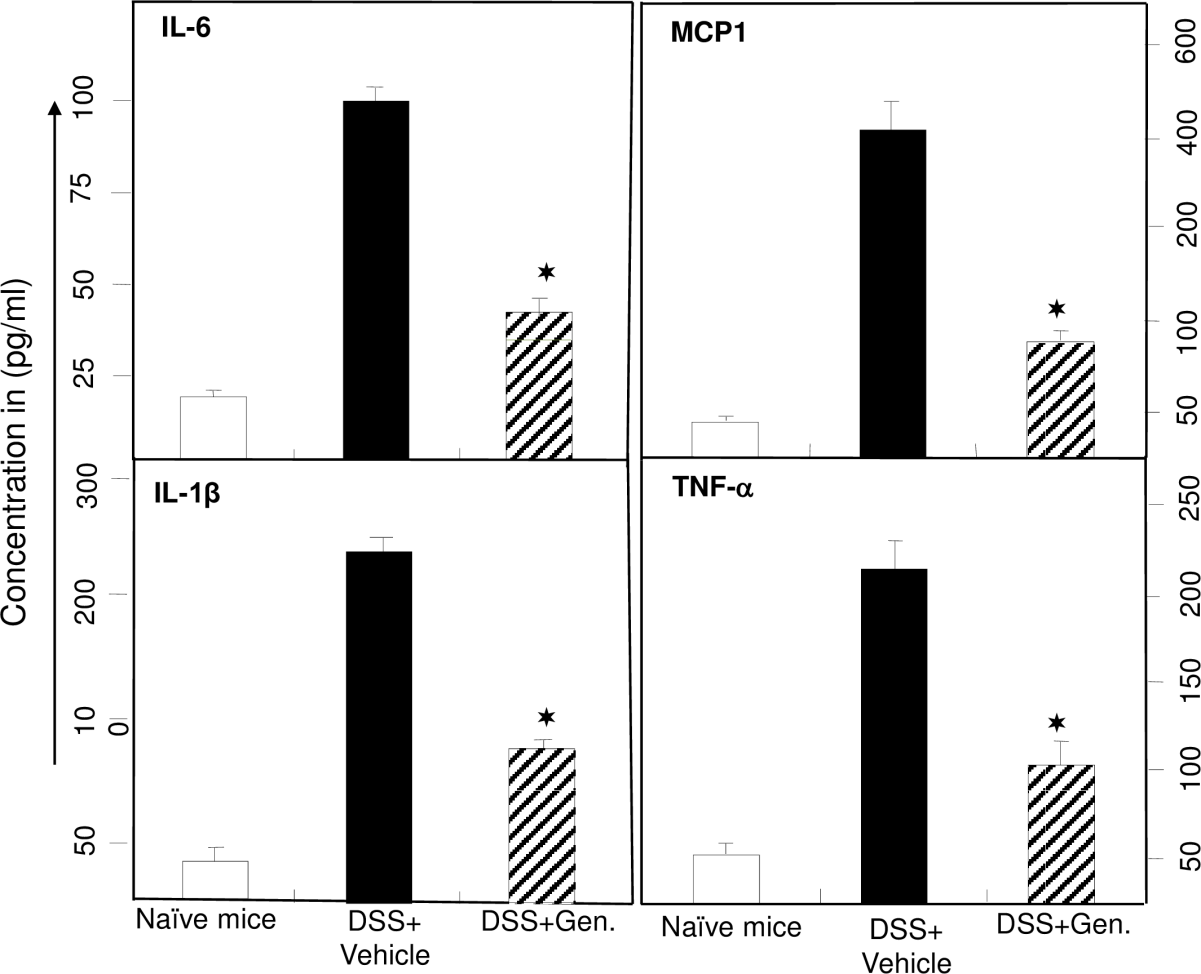
Systemic levels of IL-6, TNF-𝛂, IL-1β, and MCP-1 are reduced in groups that received genistein. After sacrifice, as described in Fig 1, serum levels of IL-6, TNF-α, IL-1β, and MCP-1 were determined with a Bio-Rad ELISA multiplex kit, which is capable of detecting >15 pg/ml of these analytes. The data presented are the mean concentrations of IL-6, TNF-α, IL-1β, and MCP-1 ± SEM from three separate experiments. Asterisks (✶) indicate statistically significant differences (p < 0.01) between DSS alone and the DSS+genistein treated group.

Increases in the level of TNF-α, IL-6 and IL-1β during IBD and experimental colitis have been well documented [19, 20]. Several clinical and experimental colitis studies have shown the upregulation of monocyte chemoattractant protein (MCP)-1 in mucosal tissues [21]. Therefore, we next determined whether, an increase in systemic cytokine concentrations that are characteristic of the disease occur in this model of colitis. We found that serum IL-6, TNFα, MCP-1 and IL-1β is increased in vehicle treated DSS induced mice (Fig 5). Further, genistein treated mice show a significant decrease (P <0.05) in all of these systemic cytokines as compared to vehicle treated mice (Fig 5). These data clearly indicate that genistein treatment leads to a reduction in the levels systemic inflammatory cytokine in DSS induced colitis and may explain, at least in part, the improved clinical outcomes observed in these mice.

### Genistein affects DCs, IL-10 expressing CD4^+^ T cells and M2 macrophages

**Fig 6:**
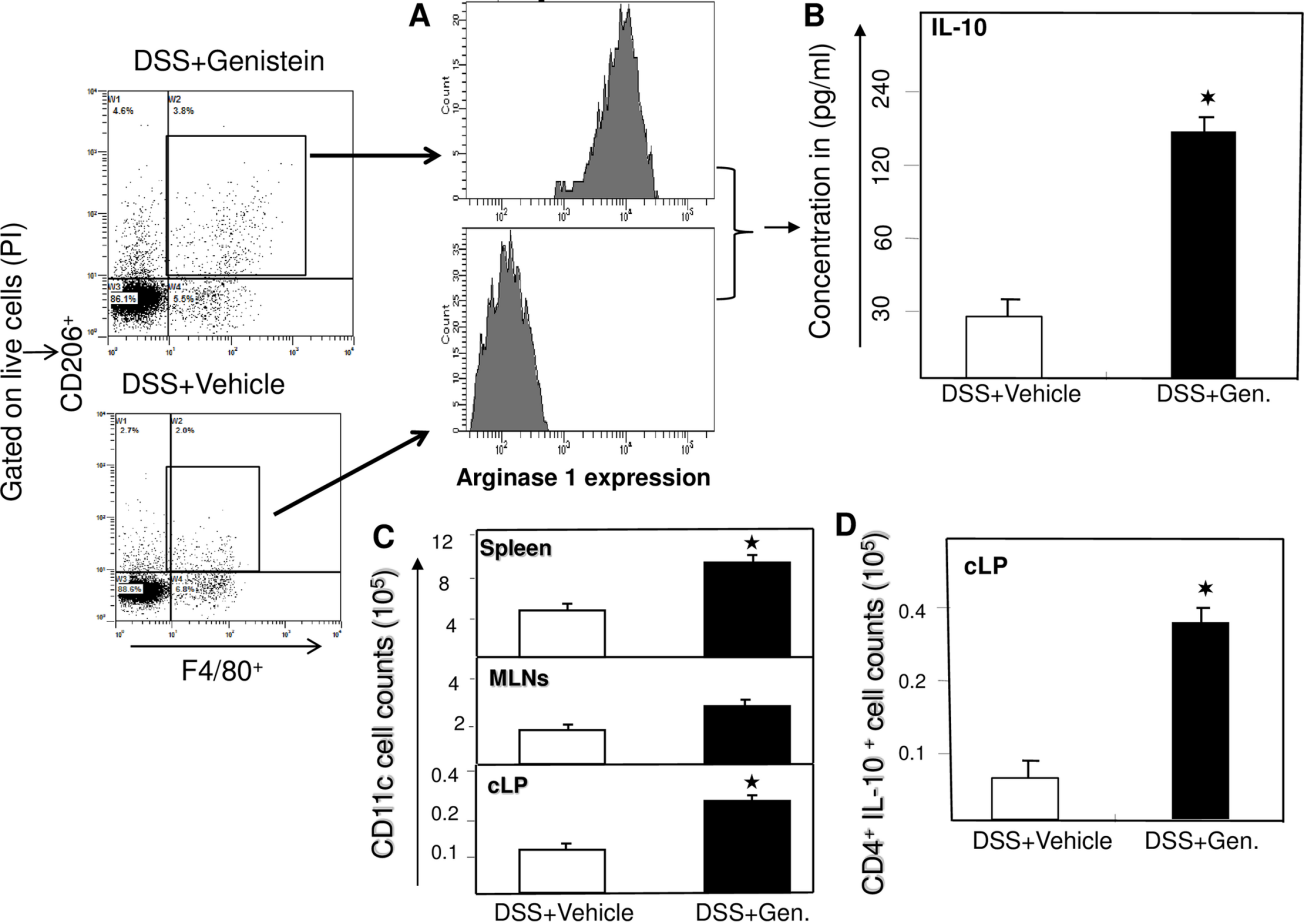
Genistein induces DCs, IL-10 expressing CD4^+^ T cells frequency, and M2 phenotypes. Characterization of genistein induced sorted purified F4/80+CD206 populations after DSS induction. M2 cells were sorted from spleen isolated from the DSS induced mice that received genistein (A upper panel) or vehicle (A lower panel) analyzed for expression of ARG-1 by flow cytometry. M2 cells from two groups were sorted and cultured *in-vitro* for 24 hours. IL-10 levels were measured from the culture supernatant by Bioplex analysis. Changes in the numbers of DCs, (Spleen, MLN and cLP) and IL-10 expressing CD4^+^ T cells (cLP) were enumerated from these groups. Data shown are from a representative experiment; three independent experiments involving six mice/group yielded similar results. The statistical comparisons were made between various groups using ANOVA and Student’s t-test as indicated in the figure and p<0.05 were indicated with an asterix (✶).

It has been shown that DCs efficiently take up antigen and present to naïve T cells, resulting in T-cell activation during colitis development. Further, production of IL-10 is an important self-regulatory function for CD4^**+**^ T lymphocytes. Therefore, next we examined the changes in number of DCs and IL-10 producing CD4^**+**^ T cells after genistein treatment. We found that both DCs and IL-10 producing CD4^**+**^ T cells number increased after genistein treatment (Fig 6 panel B and D) as compared to vehicle. We further functionally characterize the M2 macrophages, by sorting (BD Aria II; >90% purity) and *in-vitro* culture of purified M2 cells induced by genistein and vehicle. We noticed that these genistein induced M2 macrophage highly express ARG-1 (Fig 6 panel B) as compared to vehicle. Further, IL-10 levels increased in M2 cells as compared to M1 sorted purified cells after 24 hours *in-vitro* cultures (Fig 6 panel B). These data clearly indicate that genistein treatment leads to a induction of both DCs and IL-10 producing CD4^**+**^ T cells and induces the expression of ARG-1 and IL-10 levels may explain the improved clinical outcomes observed in these mice.

## Discussion

Macrophages undoubtedly provide important protection against harmful local antigens such as those that cause intestinal inflammation and specific macrophage-targeted therapies are becoming a first step in these treatments [22]. Clinical studies have shown that soy-based foods, which have been eaten for centuries in Asian countries, have potential benefits, including lowering the incidence of coronary heart disease, atherosclerosis, and type-2 diabetes [7–9]. Genistein have also been shown to be beneficial in antigen-immunized, allergic, and autoimmune disease models [10–12]. Soy is also an important mediator of gastrointestinal inflammation [13]. In the present study, we investigated the role of genistein in the protection of DSS induced experimental colitis. We found that genistein treatment ameliorated DSS-induced colitis and mice show a decline in associated symptoms (i.e. increased body weight). At the cellular level, genistein treated mice exposed to DSS treatment exhibited a reduction in inflammatory cytokines, decreased M1 and increased M2 macrophage frequency, which is associated with reduced histological disease severity and inflammation.

It has been shown that in experimental models of colitis apart from macrophages, T cells also play a role in induction of the disease. To this end, intestinal damage associated with IBD is known to be a consequence of T cell-mediated injury [23]. Previous studies from our laboratory suggest that T cells expressing CXCR3, a chemokine results in development of colitis [24]. In the present study, we noticed a decrease in T cells (both CD4 and CD8) in the spleen and cLP of genistein treated mice as compared to wild-type mice following DSS exposure. In contrast, we noticed a significant (P < 0.05) increase of T cells in MLNs after genistein treatment. This result on T cell corresponds with our earlier study on natural compounds resveratrol using similar DSS induced model of colitis [14]. These results suggest that genistein treatment also mitigates T cells in part that result in a decrease in the frequency of these cells at systemic and effector site (colon). In contrast, we noticed an increase in these cells at inductive sites (MLNs), ultimately providing protection from advanced development of experimental colitis.

The intestine is exposed to prominent effector cells of both innate and adaptive immune responses that control inflammation. In response to various signals stimulated by toll like receptors (TLRs) and IFN-γ, macrophages are skewed toward the M1 or M2 phenotype [25, 26]. M1 macrophages are associated with the initiation and continuation of inflammation; M2 macrophages are associated with the resolution or amelioration of chronic inflammation [27]. As important early mediators of protective immunity, macrophages are present in distinctly elevated numbers in the mucosa of patients with both CD and UC [6]. In the present study we noticed that genistein reduces the M1 (CD11b+ CD11c) and increases M2 (F4/80+CD206) percentage and number as compared to vehicle control. These results clearly suggest that genistein mediates colitis by skewing M1 towards M2 polarization and reduces inflammation. To this end, it has been shown that genistein suppresses the inflammatory response, morphology, and G2/M cell cycle arrest in RAW 264.7 macrophages [28, 29]. Genistein also inhibits nitric oxide and prostaglandin E2 pathways in lipopolysaccharide-induced macrophage [30]. Further, genistein also reduces the inflammatory response in delayed type hypersensitivity (DTH) reaction predominantly mediated by T cells/macrophages [12]. To be more precise, genistein has been shown to reduce chronic Trinitrobenzenesulfonic acid (TNBS) induced colitis in rat through cyclooxygenase-2 (COX-2) and myeloperoxidase (MPO) inhibition [31].

It has been shown by well defined novel gating strategy that differential expression of CD64 allows the unequivocal identification of monocytes, DCs and macrophages in the intestine of mice and humans in both healthy and inflamed conditions in T cell induced model of colitis [32]. This might be better strategy to functionally characterize macrophage phenotypes in the gut under both normal and inflamed condition. To caveat this, we sorted the M2 population after genistein treatment and further characterize by the expression of ARG-1 and IL-10 levels in these cells and data clearly support the notion. Taken together, our study highlights that genistein skews the polarization of M1 towards the M2 macrophages phenotype and reduces the inflammation in experimental colitis.

The level and expression of TNF-α and IL-6 is increased in several models of colitis, including in IBD patients [15, 33–35]. Similarly, IL-1β infiltrating cell expression increased in CD patients as well as in various experimental models of colitis [36–38]. Studies have shown the critical role of monocyte chemoattractant protein (MCP)-1 in experimental colitis [21]. Here we demonstrate a decreased level of TNF-α, IL-6, IL-1β and MCP-1 in the serum of DSS induced mice that received genistein as compared to vehicle treated mice. Taken together, results suggest that genistein suppresses systemic cytokine levels to reduce the colitis symptoms.

Genistein has been now shown to mediate various other pathways to reduce inflammation and cardiovascular diseases. Recently, it has been shown that genistein acts as anti-hypertensive agent in different experimental model by lowering vascular resistance and potentiating vasodilator mechanism [39]. In other study it has been shown that genistein reduces tyrosine kinase expression to reduce inflammation [40]. However, due to limited studies and clinical trials on genistein, further investigations are needed before definitive conclusion on mechanistic of protection in various disease models.

In summary, we have demonstrated that mice received genistein are protected from DSS-induced colitis. It is likely that genistein mediates this effect through multiple pathways including diminished both percentage and number M1, increased M2 macrophages, T cells lessening from cLP, reduced systemic inflammatory cytokine from mucosal organs. This reduction in cytokine might be due to reduction in numbers and percentage of T cells and M1 macrophages. These combined effects are likely to be responsible for the reduced severity of colitis in genistein treated mice. In summary, genistein affects T cells, skewing M1 towards M2 phenotype and mediates systemic cytokine in part to reduce colitis severity. However, a more detailed study is necessary prior to drawing any firm conclusions on how genistein induced M1 and M2 polarization and affects function to mediate colitis.

## Supporting information

S1 File Genestin 03-30-18.pptx. (PPTX)
