# Supplementary material for: Genistein induces macrophage polarization and systemic cytokine to ameliorate experimental colitis
Source: PLoS One. 2018 Jul 19;13(7):e0199631. doi: 10.1371/journal.pone.0199631 (PMC6053137; doi:10.1371/journal.pone.0199631)
Supplement: S1 File — (PPTX) [file pone.0199631.s001.pptx]

## Slide 1
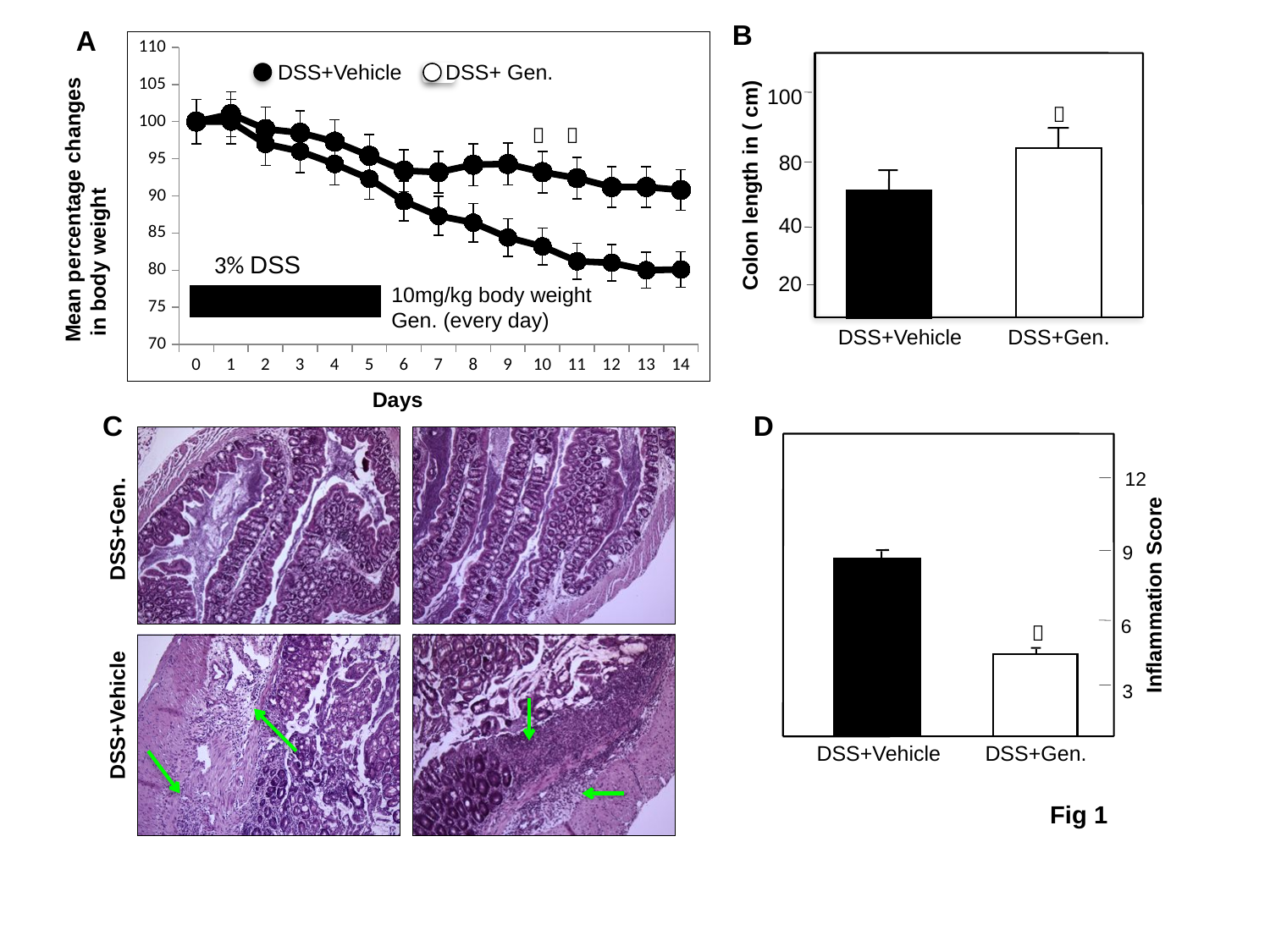

B
A
### Chart
| Category |
|---|Colon length in ( cm)
DSS+Vehicle
DSS+Gen.
### Chart
| Category | DSS | THF+DSS |
|---|---|---|
| 0.0 | 100.0 | 100.0 |
| 1.0 | 100.0 | 101.0 |
| 2.0 | 97.0 | 99.0 |
| 3.0 | 96.0 | 98.5 |
| 4.0 | 94.3 | 97.3 |
| 5.0 | 92.3 | 95.4 |
| 6.0 | 89.3 | 93.4 |
| 7.0 | 87.3 | 93.2 |
| 8.0 | 86.4 | 94.2 |
| 9.0 | 84.4 | 94.3 |
| 10.0 | 83.2 | 93.2 |
| 11.0 | 81.2 | 92.4 |
| 12.0 | 81.0 | 91.2 |
| 13.0 | 80.0 | 91.2 |
| 14.0 | 80.1 | 90.8 |DSS+Vehicle
DSS+ Gen.
100



80
Mean percentage changes
 in body weight
40
3% DSS
20
10mg/kg body weight
Gen. (every day)
Days
C
D
12
DSS+Gen.
9
Inflammation Score

6
3
DSS+Vehicle
DSS+Vehicle
DSS+Gen.
Fig 1

## Slide 2
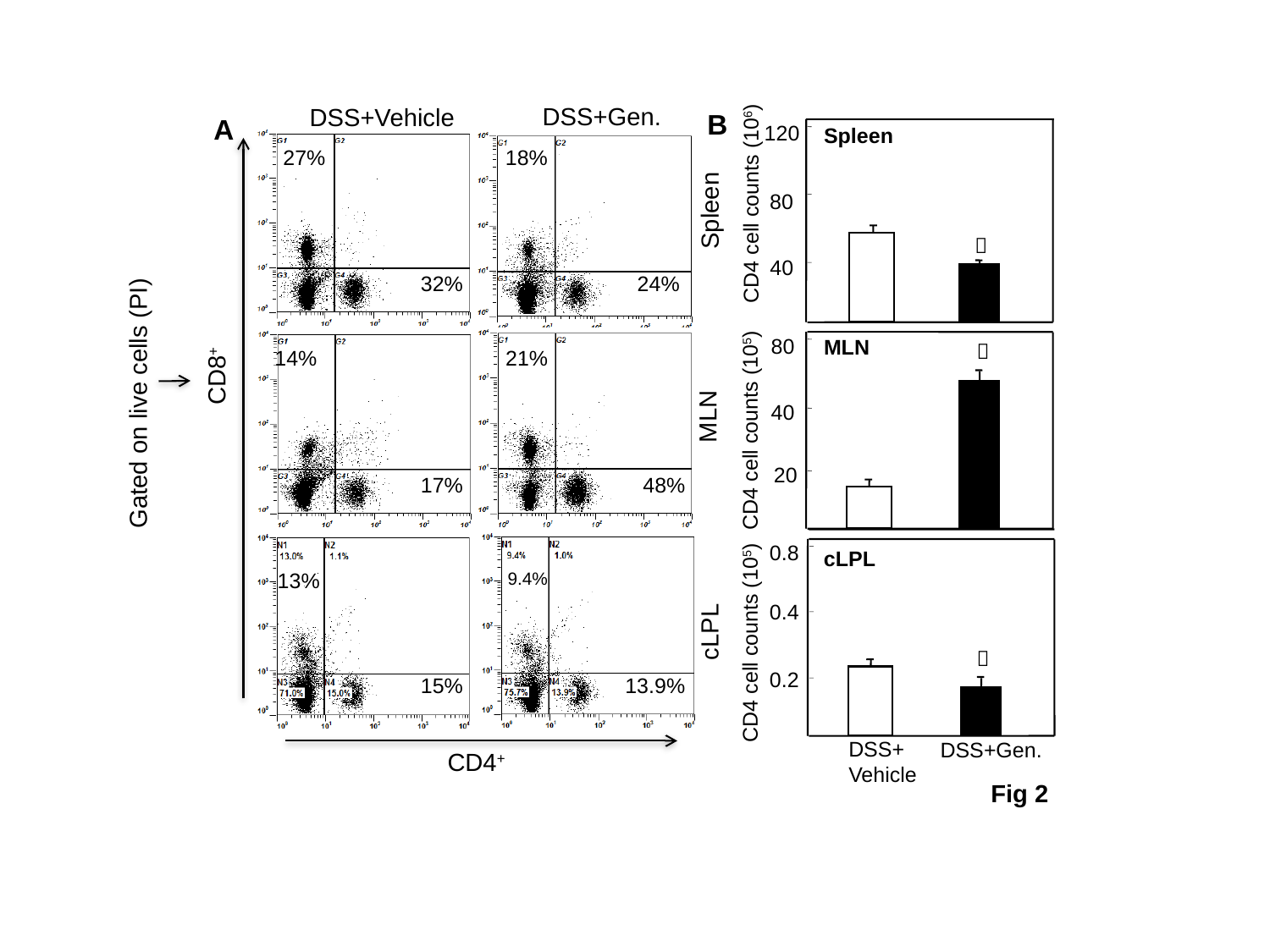

DSS+Gen.
DSS+Vehicle
B
A
Spleen
120
27%
18%
CD4 cell counts (106)
80
Spleen

40
32%
24%
MLN

80
 14%
 21%
CD8+
Gated on live cells (PI)
MLN
40
CD4 cell counts (105)
20
48%
17%
cLPL
0.8
13%
9.4%
0.4
cLPL
CD4 cell counts (105)

15%
13.9%
0.2
DSS+
Vehicle
DSS+Gen.
CD4+
Fig 2

## Slide 3
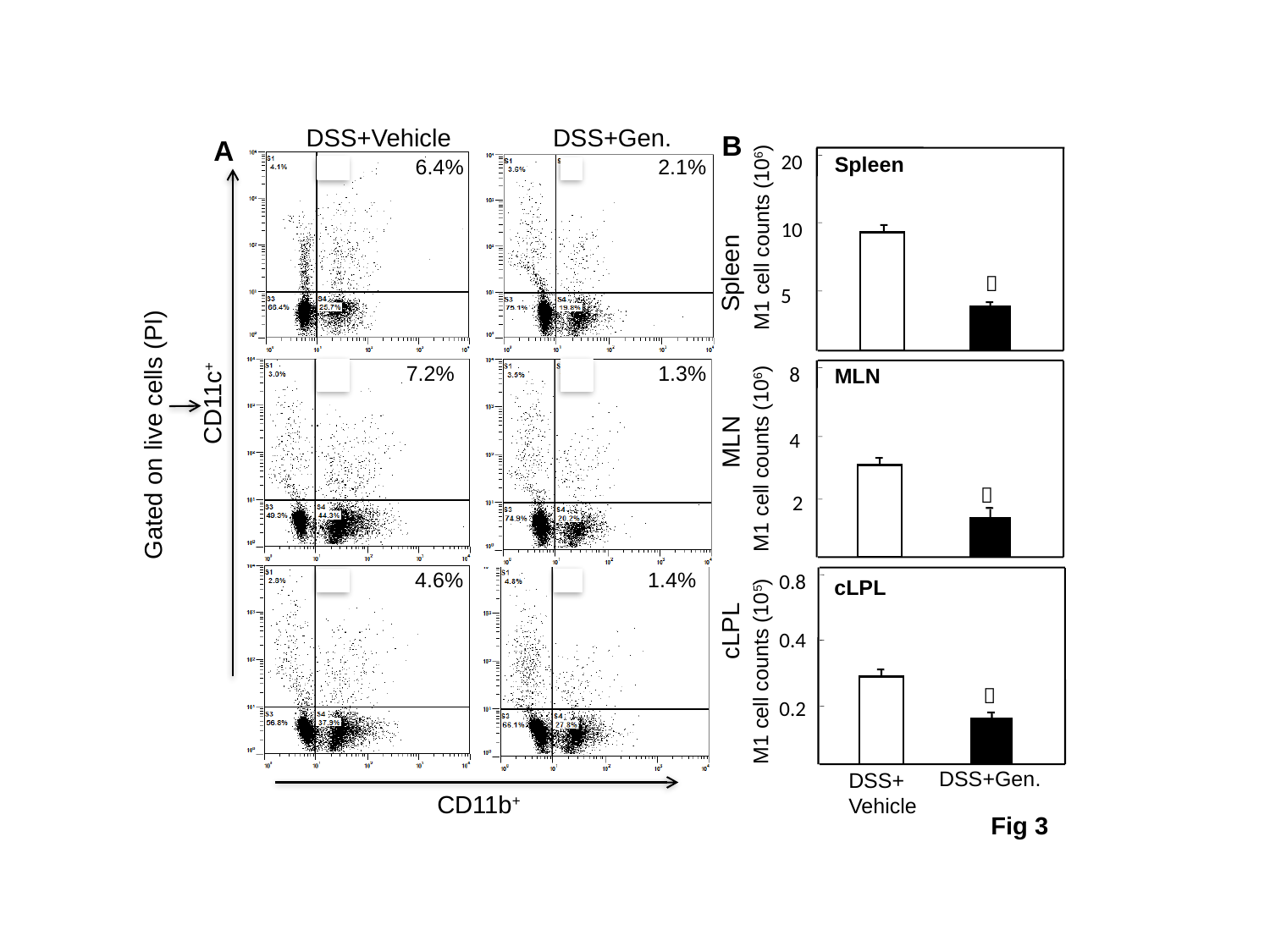

DSS+Vehicle
DSS+Gen.
B
A
Spleen
6.4%
2.1%
20
10
M1 cell counts (106)
Spleen

5
7.2%
1.3%
MLN
8
CD11c+
Gated on live cells (PI)
MLN
4
M1 cell counts (106)

2
4.6%
1.4%
cLPL
0.8
cLPL
0.4
M1 cell counts (105)

0.2
DSS+Gen.
DSS+
Vehicle
CD11b+
Fig 3

## Slide 4
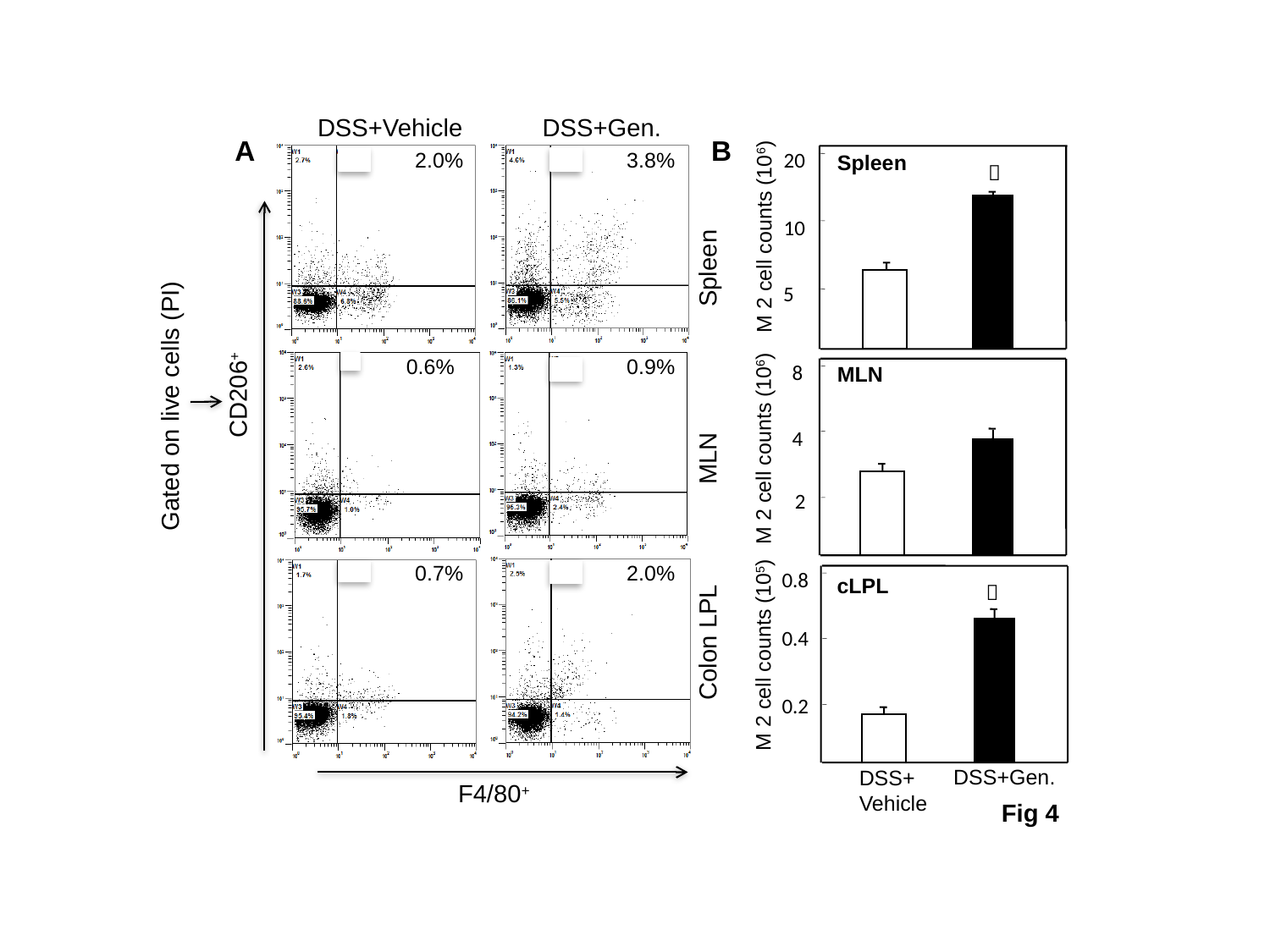

DSS+Vehicle
DSS+Gen.
2.0%
3.8%
0.6%
0.9%
0.7%
2.0%
A
B
Spleen
20

10
M 2 cell counts (106)
Spleen
5
MLN
8
CD206+
Gated on live cells (PI)
4
M 2 cell counts (106)
MLN
2
cLPL
0.8

 Colon LPL
0.4
M 2 cell counts (105)
0.2
DSS+Gen.
DSS+
Vehicle
F4/80+
Fig 4

## Slide 5
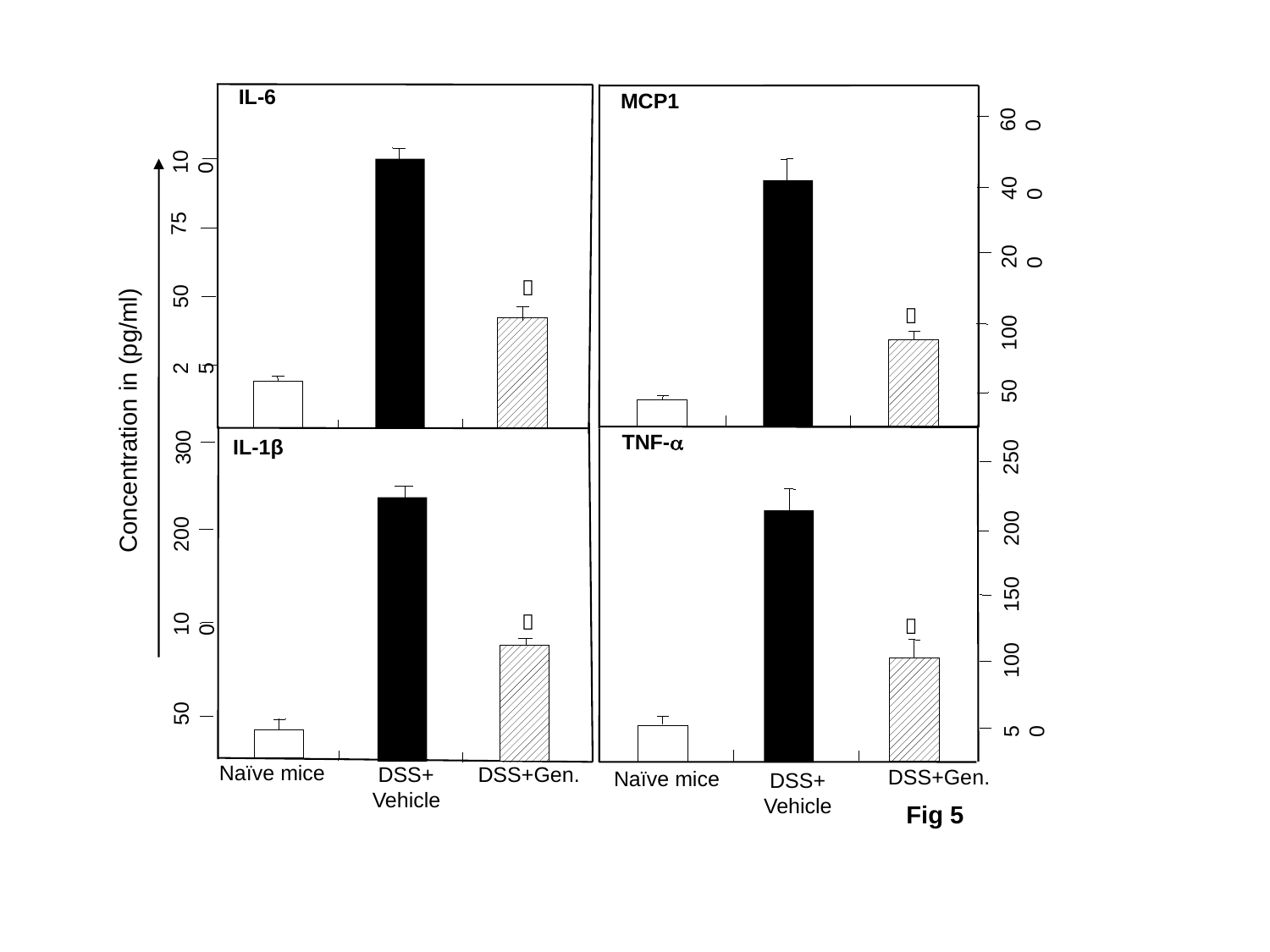

IL-6
MCP1
600
100
400
75
200

50

100
25
50
Concentration in (pg/ml)
TNF-α
300
IL-1β
250
200
200
150

100

100
50
50
 DSS+
Vehicle
DSS+Gen.
DSS+Gen.
Naïve mice
 DSS+
Vehicle
Naïve mice
Fig 5

## Slide 6
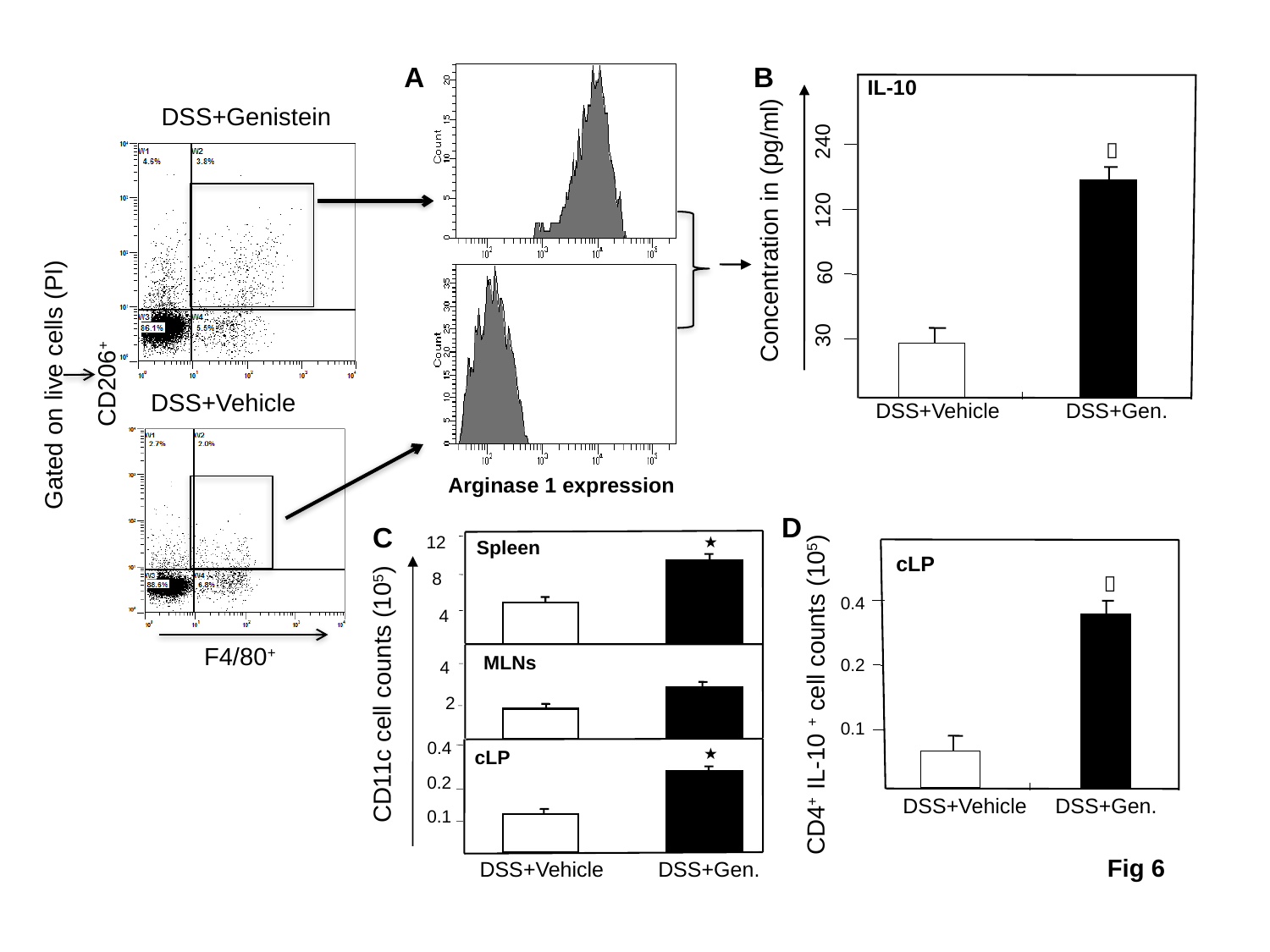

A
B
IL-10
DSS+Genistein
240

120
Concentration in (pg/ml)
60
30
Gated on live cells (PI)
CD206+
DSS+Vehicle
 DSS+Vehicle
DSS+Gen.
Arginase 1 expression
D
C
★
Spleen
12
 cLP

8
0.4
4
F4/80+
MLNs
CD4+ IL-10 + cell counts (105)
0.2
4
CD11c cell counts (105)
2
0.1
0.4
★
 cLP
0.2
DSS+Gen.
 DSS+Vehicle
0.1
Fig 6
DSS+Vehicle
DSS+Gen.
